# Assessment of Use and Fit of Face Masks Among Individuals in Public During the COVID-19 Pandemic in China

**DOI:** 10.1001/jamanetworkopen.2021.2574

**Published:** 2021-03-11

**Authors:** Xiangbin Pan, Xi Li, Pengxu Kong, Lin Wang, Rundi Deng, Bin Wen, Luoxi Xiao, Honglin Song, Yi Sun, Hongmei Zhou, Jiang Lu, Yang Wang, Qiuzhe Guo, Lin Duo, Chengye Sun

**Affiliations:** 1 Fuwai Hospital, Chinese Academy of Medical Sciences, Peking Union Medical College, Beijing, China; 2 Central China Subcenter of the National Center for Cardiovascular Diseases, Zhengzhou, China; 3 National Clinical Research Center, Fuwai Hospital, Chinese Academy of Medical Sciences, Peking Union Medical College, Beijing, China; 4 Xiangya Hospital, Central South University, Changsha, China; 5 Fuwai Yunnan Cardiovascular Hospital, Kunming, China; 6 Public Health College, Kunming Medical University, Kunming, China; 7 National Institute of Occupational Health and Poison Control, Chinese Center for Disease Control and Prevention, Beijing, China

## Abstract

**Importance:**

Face masks are recommended to prevent transmission of coronavirus disease 2019 (COVID-19); however, there is scarce evidence on their protection efficacy and ways to improve it.

**Objective:**

To determine the proportion of improper face mask use, the factors associated with face mask protection efficacy, and ways to improve efficacy.

**Design, Setting, and Participants:**

This population-based cross-sectional study was conducted in China from July to August 2020 in 5 kinds of public places. Participants included convenience samples of individuals wearing face masks and able to taste the check solution.

**Exposures:**

Demographic and socioeconomic characteristics, including sex, age, and education level; information on face mask model and the worn duration was recorded.

**Main Outcomes and Measures:**

The main outcome as airtightness, assessed by detecting face-to-face mask gaps, movement of cotton fiber placed at the face mask edges, and using a qualitative fit test with a bitter solution spray. Masks were further assessed for whether sealing the upper face mask edge with an adhesive tape strip was associated with improved face mask airtightness.

**Results:**

Among 6003 face mask wearers enrolled, the mean (SD) age of participants was 31.1 (13.7) years, and 3047 participants (50.8%) were female. The first qualitative fit test found air leakage in 2754 participants (45.9%; 95% CI, 44.6%-47.1%), which was mostly attributable to gaps at the upper face mask edge. After sealing the upper face mask edge with an adhesive tape strip, 69.7% (95% CI, 68.0%-71.5%) of masks that had exhibited leakage became airtight in the second qualitative fit test, and the rate of airtightness reached 96.2% (95% CI, 95.4%-96.8%) in a third qualitative fit test after new surgical face masks with tape on the upper edge were provided to those who had not converted initially. The tape was well tolerated; overall, 6 participants (1.2%) reported a rash and 24 participants (5.8%) reported significant discomfort.

**Conclusions and Relevance:**

In this study of face mask fit among participants in China, although most people used face masks in public places, compromised protection due to suboptimal airtightness was common. The simple approach of sealing the upper edge of the face mask with an adhesive tape strip was associated with substantially improved its airtightness.

## Key Points

**Question:**

What proportion of people in public places are wearing face masks without proper airtight seals, what factors are associated with face mask protection efficacy, and can they be improved?

**Findings:**

This cross-sectional study, including 6003 participants wearing face masks in public places, found that face mask airtightness was commonly suboptimal, mostly secondary to gaps at the upper face mask edge. Using simple and tolerable approach of sealing the upper face mask edge with an adhesive tape was associated with significant improvement of face mask airtightness.

**Meaning:**

These findings suggest that compromised protection due to suboptimal face mask airtightness was common, and use of adhesive tape to seal the upper edge was associated with easily and quickly improving the airtightness of existing masks.

## Teaser

This cross-sectional study examines the proportion of people using face masks improperly in public and the factors associated with increasing face mask airtightness.

## Introduction

Coronavirus disease 2019 (COVID-19) has spread to more than 200 countries and regions within the last year, causing a once-in-a-century health crisis with more than 90 million cases and 1.9 million deaths worldwide.^1,2^ Use of face masks has been widely recommended to reduce the risk of infection with the novel severe acute respiratory syndrome coronavirus 2 (SARS-CoV-2), the etiological agent of COVID-19, which is mainly transmitted by exhaled droplets.^3,4^ Although wearing a face mask reduces the risk of infection from 17.4% to 3.1%,^5^ a recent report documented transmission of SARS-CoV-2 during a flight despite face mask use, indicating that protection efficacy of face masks might be compromised by poor airtightness.^6^

There currently are scarce data on the airtightness of face masks. Recent surveys in US hospitals documented that airtightness of face masks, including N95 respirators, used by health care professionals was commonly suboptimal.^7,8^ For the disposable medical and surgical masks that are commonly used among community dwellers, the factors affecting face mask protection efficacy and ways to improve it remain undetermined.

Because of the urgent need to mitigate the COVID-19 pandemic, which has continued to spread with no end in sight,^9^ we investigated airtightness, a key indicator associated with protection efficacy, of commonly used face masks in a variety of public places across China. Based on the results, we developed and tested a simple way to improve airtightness among community dwellers.

## Methods

The ethics committee at Fuwai Yunnan Cardiovascular Hospital approved this study. All enrolled participants provided written informed consent. This study followed the Strengthening the Reporting of Observational Studies in Epidemiology (STROBE) reporting guideline.

### Study Design and Population

We conducted a cross-sectional study to assess airtightness of face masks, followed by an intervention study assessing a way to improve it. During July and August 2020, we chose public places, such as markets, train stations, airports, hospitals, and schools in Beijing, Yunnan, Shanxi, and Jiangsu, China, and enrolled convenience samples of individuals aged older than 6 years who were not pregnant, did not have severe respiratory tract infections or asthma, and were able to taste the check solution before face mask assessment.

### Data Collection and Variables

A paper-based questionnaire was used to collect the participants’ demographic and socioeconomic characteristics, including sex, age, and education level (classified as middle school or less, junior college, or college or higher); participant’s face mask model (N95 or KN95 [Chinese equivalent of N95; eTable 1 in the Supplement] respirator, surgical face mask, disposable medical face mask, cotton face mask, and other types, including antidust masks and activated charcoal masks), and the duration for which it had been worn. The Chinese disposable medical face mask standard is similar to the surgical mask except for lack of resistance to penetration by synthetic blood and submicron particulate filtration efficiency (eTable 2 in the Supplement).

Investigators conducted face mask airtightness testing in 3 steps: first, face masks were observed to identify potential gaps between face mask edges and participant’s face; second, cotton fibers were placed at all face mask edges to check whether they moved when participants were asked to take a deep breath (eFigure 1 in the Supplement); and third, a qualitative fit test was conducted using the FT-30 Qualitative Fit Test Kit, bitter version (3M) (eFigure 2 in the Supplement).

Before the qualitative fit test,^10^ the taste threshold was screened for each participant to verify their ability to taste the bitter test solution. Participants were instructed to put on a hood while breathing with their mouth open and their tongue partially extended. The investigator then sprayed the threshold check solution into the enclosure by inserting a nebulizer into the hole in the front of the enclosure, and asked participants whether the solution could be tasted. Participants who could not taste the test solution were considered ineligible for the study.

For eligible participants, formal testing following a similar process was then conducted. After the spray, the participants were asked to execute few tasks for 7 minutes, such as talking, taking deep breaths, or bowing their heads. Investigators would then ask participants whether they could taste the check solution (positive result) or not (negative result).

### Intervention and Measurement

**Figure 1.**
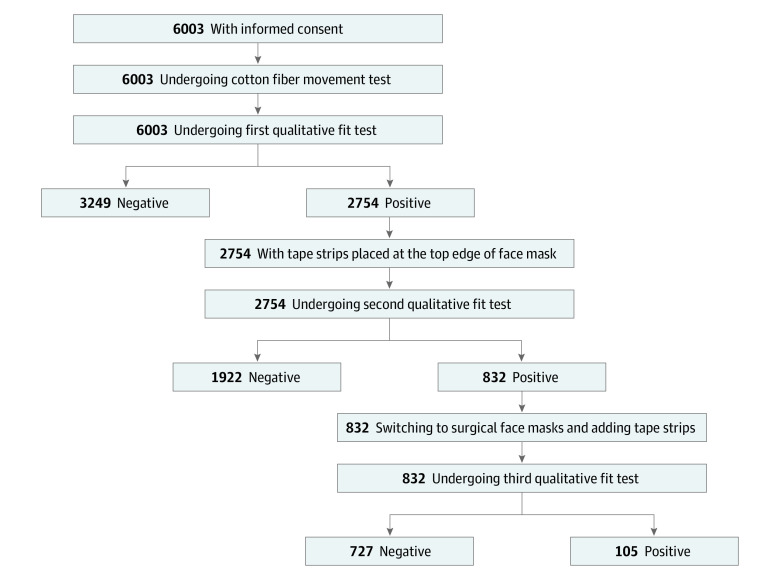
Flowchart of Participant Enrollment and Tests

For the participants with positive results for the qualitative fit test, a tape strip (Single-side Tape 1538-1; 3M) was used to seal the upper edge of the participant’s face mask against the face (eFigure 3 in the Supplement) to improve airtightness. The sealing area was centered around the bridge of the nose, slightly exceeding the distance between pupils. After correctly sticking the tape, the participants were instructed to put on the hood and again underwent the qualitative fit test. If the second qualitative fit test yielded a positive result, participants were asked to wear a new surgical face mask, with a similarly applied tape strip. After putting on the hood, a third qualitative fit test was conducted (Figure 1).

Among participants enrolled in hospitals in August 2020, investigators conducted a follow-up interview as they were leaving the hospital after a clinical visit. Simple questions on tolerability of the tape were asked, including potential skin symptoms and discomfort.

### Statistical Analysis

In separate public places, we determined the sample size required to achieve a descriptive precision of 5% with an α = .05 (ie, a 95% CI width of 10%) for the primary outcomes, which was the rate of positive results in the first qualitative fit tests and the rate of participants with positive test results who had negative results in the second qualitative fit test. Because assuming rates of 50% would require the largest sample size, we aimed to enroll 1000 participants in each of the 5 types of public places. Participants’ characteristics and results of tests and interventions are described as frequencies and percentages for categorical variables and means and SDs or median with interquartile ranges (IQRs) for continuous variables. χ^2^ tests were used to assess bivariate associations, and a logistic regression was used to identify factors independently associated with face mask airtightness. Missing values were not imputed. Participants lost to follow-up were excluded from the tolerability assessment. All comparisons were 2-tailed, with a *P* < .05 considered statistically significant. All statistical analyses were performed with the SPSS statistical software version 21.0 (IBM).

## Results

### Participant Characteristics

A total of 6003 participants were enrolled, including 1042 (17.4%) in schools, 1318 (22.0%) in train stations, 1099 (18.3%) in hospitals, 1274 (21.2%) in airports, and 1270 (21.2%) in public markets. The mean (SD) age of participants was 31.1 (13.7) years, and 3047 participants (50.8%) were female. Education level was college or above in 1838 participants (30.6%); junior college in 2160 participants (35.1%); and middle school or below in 2059 (34.3%) (Table 1).

**Table 1.** Baseline Characteristics of Study Participants

Variable	Participants, No. (%)					
	School (n = 1042)	Train station (n = 1318)	Hospital (n = 1099)	Airport (n = 1274)	Market (n = 1270)	Total (n = 6003)
Age, y						
Mean (SD)	19.5 (8.1)	32.3 (13.5)	32.4 (12.5)	34.4 (13.6)	34.7 (13.5)	31.1 (13.7)
<20	921 (88.4)	125 (9.5)	51 (4.6)	18 (1.4)	1 (<0.1)	1116 (18.6)
20-40	81 (7.8)	857 (65.0)	781 (71.1)	869 (68.2)	880 (69.3)	3468 (57.8)
>40	40 (3.8)	336 (25.5)	267 (24.3)	387 (30.4)	389 (30.6)	1419 (23.6)
Male	556 (53.4)	694 (52.7)	436 (39.7)	658 (48.4)	612 (51.8)	2956 (49.2)
Education level						
Middle school or below	281 (27.0)	468 (35.5)	323 (29.4)	503 (39.5)	484 (38.1)	2059 (34.3)
Junior college	571 (54.8)	438 (33.2)	305 (27.8)	383 (30.1)	409 (32.2)	2160 (35.1)
College or above	190 (18.2)	412 (31.3)	471 (42.9)	388 (30.5)	377 (29.7)	1838 (30.6)
Face mask type						
Disposable medical	476 (45.7)	620 (47.0)	246 (22.4)	578 (45.4)	520 (40.9)	2440 (40.6)
Surgical	498 (47.8)	606 (46.0)	308 (28.0)	606 (47.6)	678 (53.4)	2696 (44.9)
N95 or KN95 respirator	11 (1.1)	16 (1.2)	510 (46.4)	12 (0.9)	15 (1.2)	564 (9.4)
Cotton	16 (1.5)	38 (2.9)	10 (0.9)	30 (2.4)	25 (2.0)	119 (2.0)
Other^a^	41 (3.9)	38 (2.9)	25 (2.3)	48 (3.8)	32 (2.5)	184 (3.1)
Notable gaps between face and face mask	24 (2.3)	257 (19.5)	123 (11.2)	163 (12.8)	157 (12.4)	724 (12.1)
Cotton fiber movement on face mask during deep breath						
Edges	263 (25.2)	503 (38.2)	253 (23.0)	415 (32.6)	387 (30.5)	1821 (30.3)
Upper edge	248 (23.8)	401 (30.4)	214 (19.5)	358 (28.1)	336 (26.5)	1557 (25.9)
Duration of wear, median (IQR), d	2 (1-4)	2 (1-4)	2 (1-3)	2 (1-3)	2 (1-3)	2 (1-3)

Abbreviation: IQR, interquartile range.

^a^ Other types include antidust or activated charcoal mask.

At enrollment, all participants were wearing face masks: 564 participants (9.4%) were wearing N95 and KN95 respirators, 2696 participants (44.9%) were wearing surgical masks, 2440 participants (40.6%) were wearing disposable medical masks, and 119 participants (2.0%) were wearing cotton masks. These face masks had been used for a median (IQR) of 2 (1-3) days before enrollment, and for longer than 3 days in 1439 participants (24.0%). N95 and KN95 respirators were more commonly used among participants who were aged 20 to 40 years, were female, had a college education, or were recruited in hospitals. Participants with college education had face masks with a shorter duration of use (median [IQR], 2 [1-3] days vs 2 [1-4] days in middle school or below and 2 [1-3] days in junior college; *P* = .02).

### Airtightness and Related Factors

During the tests for air tightness, 724 participants (12.1%; 95% C, 11.3%-12.9%) were observed to have notable gaps between their face and face mask. Additionally, in 1821 participants (30.3%; 95% CI, 29.2%-31.5%), the cotton fiber moved at the edges of the face mask during breathing, with movement for 1557 participants (85.5%) at the upper edge. Overall, the first qualitative fit test yielded positive results among 2754 participants (45.9%; 95% CI, 44.6%-47.1%). The highest positivity rate was in train stations (49.3%; 95% CI, 46.9%-52.3%), and the lowest rate was in hospitals (37.3%; 95% CI, 34.5%-40.2%) (*P* < .001).

In multivariate analysis, poor face mask airtightness was associated with the type of face mask and the duration it had been worn, with better results among N95 and KN95 respirators (adjusted OR, 0.32; 95% CI, 0.25-0.39; *P* < .001) and surgical face masks (adjusted OR, 0.83; 95% CI, 0.74-0.93; *P* = .001) compared with disposable medical face masks. Face mask airtightness deteriorated with duration of use, with an adjusted OR of 1.31 (95% CI, 1.26-1.36; *P* < .001) per additional day of wear. Age, sex, and education level of participants were not independently associated with face mask airtightness (Table 2).

**Table 2.** Factors Associated With Poor Airtightness

Factor	Multivariable OR (95% CI)	*P* value
Age group, y		
<20	1 [Reference]	NA
20-40	1.00 (0.87-1.16)	.97
>40	1.04 (0.87-1.23)	.70
Sex		
Male	1 [Reference]	NA
Female	1.04 (0.94-1.16)	.44
Education level		
≤Middle school	1 [Reference]	NA
Junior college	1.03 (0.90-1.18)	.64
≥College	1.05 (0.91-1.21)	.51
Face mask type		
Disposable medical	1 [Reference]	NA
Surgical	0.83 (0.74-0.93)	.001
N95 or KN95 respirator	0.32 (0.25-0.39)	<.001
Cotton	1.25 (0.86-1.82)	.25
Other ^a^	0.94 (0.69-1.27)	.68
Duration of face mask use, per 1-d increase	1.31 (1.26-1.36)	<.001

Abbreviations: OR, odds ratio; NA, not applicable.

^a^ Other types include antidust or activated charcoal mask.

### Using a Tape Seal at the Upper Face Mask Border

**Figure 2.**
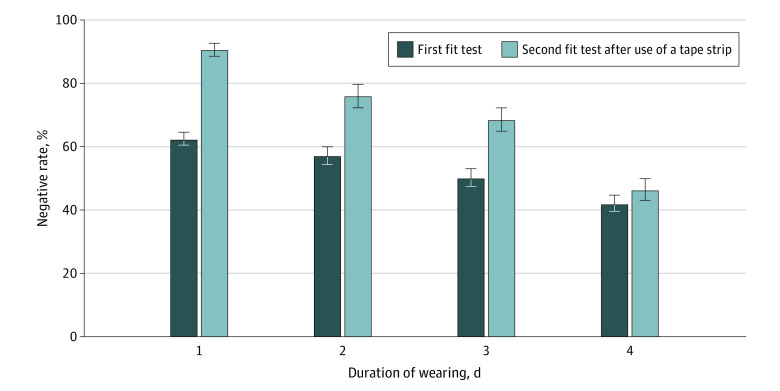
Rate of Negative Results in the First and Second Fit Tests Error bars indicate 95% CI.

Among 2754 participants with positive results in the first qualitative fit test, 1922 participants (69.7%; 95% CI, 68.0%-71.5%) had negative results in the second qualitative fit test after placing a tape at the upper edge of their face masks; the rate of conversion decreased with duration of face mask use (Figure 2). After adjusting for face mask type and duration of face mask use, the conversion rate did not differ between groups by age (compared with age <20 years, age 20-40 years: OR, 0.94; 95% CI, 0.72-1.21; age >40 years: OR, 0.80; 95% CI, 0.60-1.08), sex (women vs men: OR, 0.87; 95% CI, 0.72-1.05), or education level (compared with ≤middle school, junior college: OR, 1.19; 95% CI, 0.94-1.50; ≥college: OR, 1.26; 95% CI, 0.97-1.64).

For each of the 832 participants with positive results in the second qualitative fit test, we provided a new surgical face mask with tape on it, and 727 participants (87.4%) had negative results in the third qualitative fit test. Thus, the overall conversion rate for the second and third qualitative fit test was 96.2% (95% CI, 95.4%-96.8%).

After using the tape seal, 502 participants were followed up for a median (IQR) 4 (3-5) hours. A total of 6 participants (1.2%) reported rash on the contact site. Most participants commented that the tape was tolerable; 24 participants (4.8%) reported that the tape was significantly uncomfortable.

## Discussion

This cross-sectional study conducted in various public places in China found that despite a high rate of face mask use, face mask airtightness was commonly suboptimal, mostly secondary to gaps at the upper face mask edge. We also found that the simple and tolerable approach of sealing the upper face mask edge with an adhesive tape was associated with significantly improving the airtightness of commonly used face masks.

Our study has extended the literature in several ways. First, this study reported that the airtightness of face masks in community populations was suboptimal, which may be a reason why many countries have reported a great number of COVID-19 cases despite face mask use, including among medical staff and passengers on public transit.^6,11,12,13^ It is common that face masks are worn beyond their expiration time (typically 4 hours),^14,15,16,17^ which could be one of the major risks. Improper face mask use is a challenge to pandemic control in public places.^3,18,19,20^

Second, we identified the major factors associated with airtightness of face masks. In addition to the types and duration of use of face masks, the gaps at the upper edge should be noticed as a major vulnerability. It is difficult to fit the upper edge of face masks against the nose and face, particularly during talking and coughing, which could generate gaps that substantially compromise the protection efficacy. These problems could be considered a plausible explanation of sporadic or clustered infections, even with qualified mask use.^6,21,22,23^

Third, the airtightness of face masks was not significantly associated with participant age, sex, or education level. This, as a first-ever finding in this area, to our knowledge, was unexpected. Prior studies have reported that characteristics associated with better health literacy were positively associated with hygiene practices and health behaviors regarding prevention of infectious diseases.^24,25,26^ However, anyone could be at risk of poor face mask airtightness, regardless of their demographic and socioeconomic characteristics. This highlights the importance of improving the equipment, which can have more direct effects on protection efficacy in the general population.

Last but most importantly, based on the new knowledge on related susceptibility identified in our survey, we developed and verified a simple approach of using adhesive tape that was associated with improved airtightness of commonly used face masks. To our knowledge, this is the first large-scale assessment in real world practice. The assessment showed substantial improvement in air tightness, from 54% to 86%, for most widely used face masks. Moreover, the tape was well accepted by the participants, with rare significant discomfort, which bodes well for adoption for daily use.

Our study has implications for public health, as the pandemic has been becoming a new normal.^27^ Policies on personal protection, like face masks, should be more detailed and precise: they should not only recommend but also to underscore the right way to use face masks. To improve protection, it is imperative to improve the face mask. To this end, sealing the upper edge gap with adhesive tape might easily and quickly improve the air tightness of existing masks, with negligible cost. The tape can be reattached after removing the masks to eat and drink. Furthermore, the tape can be instantly changed if it is not adhesive. Currently, global manufacturers produce more than 100 million face masks daily. Governments could consider modifying the related industry standards and product specifications without significant changes in the current production progress. Moreover, manufacturers could develop better materials for the tape to reduce risk of allergy and skin discomfort; for example, double-sided tape could be used on the inside of the upper edge of masks.

### Limitations

This study has some limitations. First, the survey was not based on a random sampling design, which limited the estimation of national or regional proportion of proper face mask use, but would not affect the identification of factors associated with airtightness or assessing the salutary outcomes associated with tape use. Second, we used airtightness of face masks as a proxy of their protection efficacy, which could also be affected by filtering capacity and other factors. Thus, the improved airtightness associated with tape may only partly address the problems in protection. However, air tightness is the prerequisite for protection efficacy. Third, the qualitative fit test is considered the criterion standard to assess air tightness^10^; however, the study relied on participants’ self-report, which could be affected by their taste sensitivity and test adherence. Fourth, the qualitative fit test may not fully reflect the protection efficacy against viruses, because it mainly detects the filtration efficacy for droplets, but not for aerosols. However, the qualitative fit test is the preferred practical test method in real world. Also, considering that current evidence suggests that droplets are the dominant transmission mode of SAS-CoV-2,^28,29,30^ the assessment based on the qualitative fit test can generate instructive evidence for personal protection. Fifth, this study did not assess whether facial hair was associated with proper face mask use or airtightness. Nevertheless, most people in China do not have long facial hair that can interfere with the protection efficacy of face masks, which was also demonstrated by our result that sex was not associated with the improper face mask use. Sixth, this cross-sectional study was conducted in 1 country. However, large sample size decreases margin of error in results and multiple locations and populations may increase its generalizability.

## Conclusions

In this cross-sectional study, although most people in China used face mask at public places, compromised protection due to suboptimal airtightness was common. The simple approach of using adhesive tape to seal the upper edge was associated with substantially improved protection efficacy of commonly used face masks.

## Notes

Supplement. **eFigure 1.** Cotton Fiber Movement Test **eFigure 2.** Qualitative Fit Test **eFigure 3.** Method for Placing a Tape Strip on the Face Mask **eTable 1.** Comparison Between N95 and KN95 Respirators **eTable 2.** Comparison Between Disposable Medical Masks and Surgical Masks
