# Supplementary material for: Assessment of Use and Fit of Face Masks Among Individuals in Public During the COVID-19 Pandemic in China
Source: JAMA Netw Open. 2021 Mar 11;4(3):e212574. doi: 10.1001/jamanetworkopen.2021.2574 (PMC7953274; doi:10.1001/jamanetworkopen.2021.2574)
Supplement: Supplement. — eFigure 1. Cotton Fiber Movement Test eFigure 2. Qualitative Fit Test eFigure 3. Method for Placing a Tape Strip on the Face Mask eTable 1. Comparison Between N95 and KN95 Respirators eTable 2. Comparison Between Disposable Medical Masks and Surgical Masks [file jamanetwopen-e212574-s001.pdf]

Supplemental Online Content

Pan X, Li X, Kong P, et al. Assessment of use and fit of face masks among individuals in public during the COVID-19 pandemic in China. *JAMA Netw Open*. 2021;4(3):e212574. doi:10.1001/jamanetworkopen.2021.2574

eFigure 1. Cotton Fiber Movement Test
eFigure 2. Qualitative Fit Test
eFigure 3. Method for Placing a Tape Strip on the Face Mask
eTable 1. Comparison Between N95 and KN95 Respirators
eTable 2. Comparison Between Disposable Medical Masks and Surgical Masks

This supplemental material has been provided by the authors to give readers additional information about their work.

© 2021 Pan X et al. *JAMA Network Open.*

eFigure 1. Cotton Fiber Movement Test

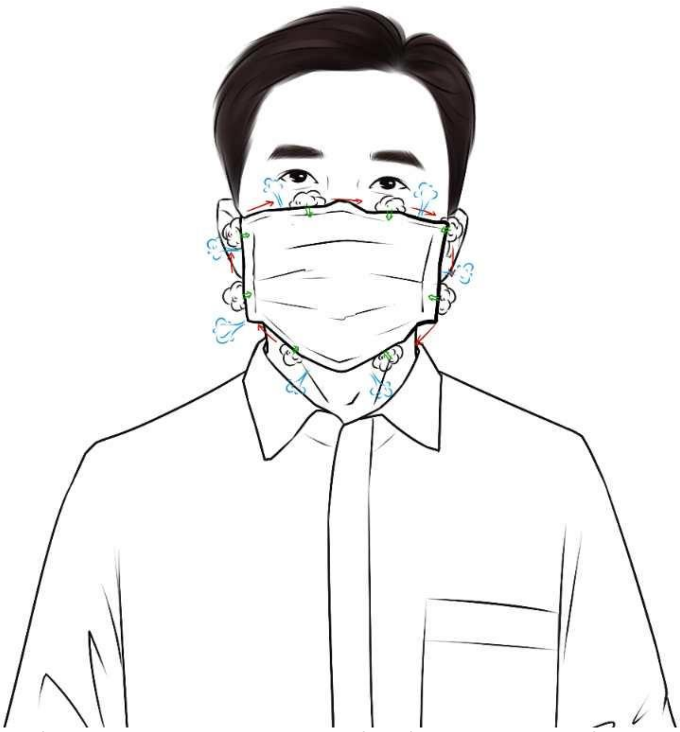

Cotton fibres were placed at each edge of the facemask to check for their movement when participants took a deep breath at investigator's request.

© 2021 Pan X et al. *JAMA Network Open.*

eFigure 2. Qualitative Fit Test

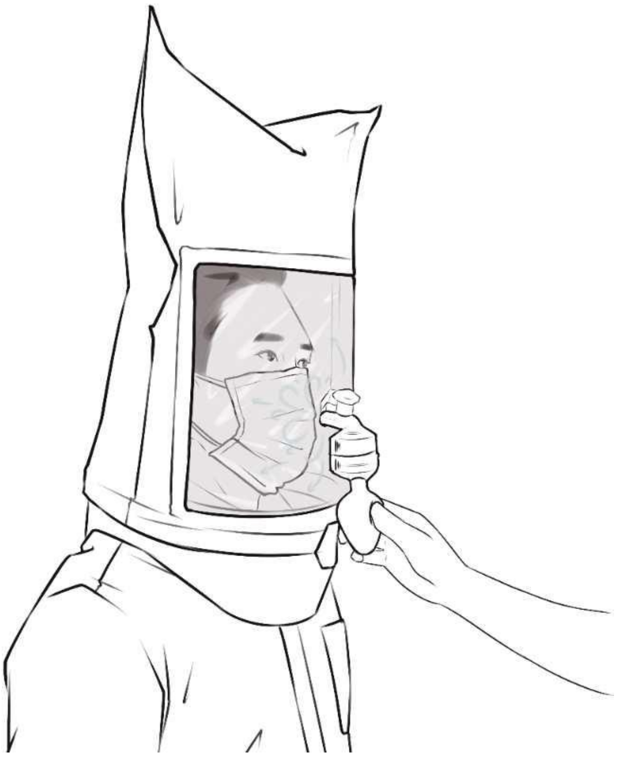

With facemasks on, participants were instructed to put on the hood, and breath with mouth open, tongue slightly extended, as the investigators sprayed FT-30 Qualitative test solution. The nozzle would be directed away from the nose and mouth of the participant. After the spray, the participants were asked to execute tasks over seven minutes, including talking, taking deep breaths, or bowing with head down. Then investigators would ask participants whether they could taste (positive) or not (negative) the check solution.

© 2021 Pan X et al. *JAMA Network Open.*

eFigure 3. Method for Placing a Tape Strip on the Face Mask

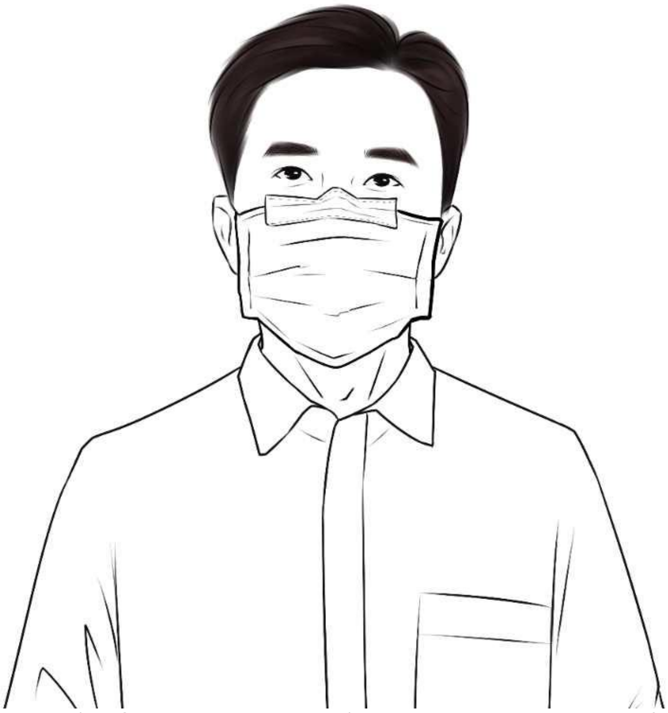

A tape strip (3M Single-side Tape 1538-1) was adhered to the top edge of the participant's face mask and skin to improve the air tightness. The sticking area was centred to the bridge of the nose. The length of tape should be longer than the distance between pupils.

© 2021 Pan X et al. *JAMA Network Open.*

eTable 1. Comparison Between N95 and KN95 Respirators

Characteristic	N95 (NIOSH-42 CRF84)	KN95 (GB 2626-2019)
Filter performance	≥95%	≥95%
Test agent	NaCl	NaCl
Flow rate	85 L/min	85 L/min
Total inward leakage	N/A	≤ 8% leakage
Inhalation resistance	≤ 343 Pa	≤ 350 Pa
Exhalation resistance	≤ 245 Pa	≤ 250 Pa
Force applied	-245 Pa	-250 Pa

© 2021 Pan X et al. *JAMA Network Open.*

eTable 2. Comparison Between Disposable Medical Masks and Surgical Masks

Characteristic	Disposable medical mask	Surgical mask
Bacterial filtration efficiency, %	95	≥95
Differential pressure, mm H_2O/cm^2	<5.0	<4.0
Sub-micron particulate filtration efficiency at 0.1 micron, %	not required	≥95
Resistance to penetration by synthetic blood, minimum pressure in mm Hg for pass result	not required	80
Flame spread	not required	Class 1

The standard of disposable medical mask is from the Pharmaceutical industry standard of the People's Republic of China YY/T 0969-2013. The standard of surgical mask is from the American Society for Testing and Materials standard ASTM F2100:19 Level 1.

© 2021 Pan X et al. *JAMA Network Open.*
